# Local Treatments in the Unresectable Patient with Colorectal Cancer Metastasis: A Review from the Point of View of the Medical Oncologist

**DOI:** 10.3390/cancers13235938

**Published:** 2021-11-25

**Authors:** Javier Torres-Jiménez, Jorge Esteban-Villarrubia, Reyes Ferreiro-Monteagudo, Alfredo Carrato

**Affiliations:** 1 Medical Oncology Department, University Hospital Ramon y Cajal, 28034 Madrid, Spain; elementjorge00@gmail.com (J.E.-V.); reyes-ferreiro@hotmail.com (R.F.-M.); 2 Medical Oncology Department, Ramón y Cajal Health Research Institute (IRYCIS), CIBERONC, Alcalá University, University Hospital Ramon y Cajal, 28034 Madrid, Spain; acarrato@telefonica.net

**Keywords:** metastatic colorectal cancer, interventional oncology in metastatic colorectal cancer, tumor ablation, liver intraarterial hepatic therapies, DEBIRI TACE, Yttrium-90

## Abstract

**Simple Summary:**

Local treatments represent a potential curative approach in the patient with liver metastases of colorectal origin. It is important for all the team involved in patient care to know and understand which patients are suitable for this approach and the expected results of these treatments. This review is intended to focus (without neglecting the technical issues of these techniques) in clinical aspects, to help other clinicians to seek the best available evidence to guide their decisions and advocate for the best possible treatment for the patient.

**Abstract:**

For patients with isolated liver metastases from colorectal cancer who are not candidates for potentially curative resections, non-surgical local treatments may be useful. Non-surgical local treatments are classified according to how the treatment is administered. Local treatments are applied directly on hepatic parenchyma, such as radiofrequency, microwave hyperthermia and cryotherapy. Locoregional therapies are delivered through the hepatic artery, such as chemoinfusion, chemoembolization or selective internal radiation with Yttrium 90 radioembolization. The purpose of this review is to describe the different interventional therapies that are available for these patients in routine clinical practice, the most important clinical trials that have tried to demonstrate the effectiveness of each therapy and recommendations from principal medical oncologic societies.

## 1. Introduction

Colorectal cancer (CRC) is the third cancer in incidence rate in adults and the second most common cause of cancer-related death in Europe [1,2,3]. Metastatic disease to the liver is present in 20% of patients with CRC at the time of diagnosis and appears in an additional 40% over the course of earlier-stage disease treated with resection. In 30% of patients, the liver is the only site of metastatic disease [4]. Liver resection or surgical metastasectomy is the best chance of cure for patients with liver metastases of colorectal cancer (LMCRC), especially in liver-limited or oligometastatic disease [5]. The number of metastases accepted as oligometastic disease that would benefit from local treatment is not well established, but the change in prognosis of these patients appears in the 8th American Joint Committee on Cancer (AJCC) staging (M1a: metastases at one site, M1b: metastases at two or more sites).

However, only up to 25% of eligible patients undergo resection because of the presence of co-morbidities or unresectability. Non-surgical local therapies, loco-regional or liver-directed therapies are significant therapies for treatment of LMCRC given the response rates to systemic chemotherapy (QT) alone [6]. Although several modern combinations of treatments (5-fluouracil (5-FU)/leucovorin (LV)/oxaliplatin (FOLFOX), 5-FU/LV/irinotecan (FOLFIRI), 5-FU/LV/oxaliplatin/irinotecan (FOLFOXIRI), targeted therapies as anti-epidermal growth factor receptor (EGFR) for RAS wild-type tumors, such as cetuximab and panitumumab and anti-vascular endothelial growth factor (VEGF), such as bevacizumab and aflibercept) have been developed recently, objective response rates (ORR) to first-line treatments for metastatic CRC range from 34% to 66%, and from 30% to 40% for second line [7].

The election of the kind of non-surgical local therapy is influenced by several factors: the size and localization of LMCRC, the local control rates achieved, the invasiveness of the technique, the local expertise regarding the use of each therapy and patients’ preferences [5]. Non-surgical local treatments in LMCRC require a multidisciplinary approach, including medical oncologists, surgical oncologists, radiation oncologists and interventional radiologists.

Non-surgical therapies in LMCRC are classified in different ways. The most useful classification is based on how the treatment is administered (Table 1). Local treatments are applied directly on hepatic parenchyma, and locoregional therapies facilitate the infusion of diverse agents into hepatic vessels. Goal of treatment is also useful to classify these therapies. It is important to note that local ablative therapies should be offered to patients in which all visible disease can be treated with a curative intent, while loco-regional therapies are usually offered to non-resectable and non-ablatable patients with LMCRC in order to improve local control along with systemic therapy, as will be discussed below.

Local ablative therapies (LAT) produce thermal damage to tissues. They include radiofrequency ablation (RFA), microwave ablation (MWA), laser ablation and ultrasound ablation, which produce damage with the increase in temperature, whereas cryoablation freezes tissue, which leads to cell death. Irreversible electroporation or percutaneous instillation of ethanol are under investigation in LMCRC.

Stereotactic body radiotherapy (SBRT) consists in a precise delivery of high doses of radiation to an extracranial target in a small number of fractions, sparing surrounding critical tissue. SBRT needs accurate immobilization, a method to manage the respiratory motion of the target and image guidance to ensure proper alignment and delivery of the radiation dose [8,9].

Locoregional therapies are distributed through the hepatic artery (embolization procedures or hepatic arterially directed therapies) for the treatment of LMCRC. These therapies include bland particulate embolization, chemoinfusion, chemoembolization and radioembolization. The health liver receives 80% of its blood supply from the portal vein and 20% from the hepatic artery but, in contrast, hepatic malignancies receive 80% of their blood supply from the hepatic artery. The dual blood supply to the liver explains the effectiveness of hepatic arterially directed therapies.

This review focuses only in two kinds of non-surgical local treatments in LMCRC: ablative local therapies and hepatic arterially directed therapies. The main clinical trials and retrospective comparations that have tried to demonstrate the effectiveness of each therapy and recommendations from principal medical oncologist societies are described.

## 2. Definition of Unresectability

Unresectable LMCRC are the patients with multiple and bilobar disease for whom R0 resection cannot be performed, leaving at least 20–25% of total liver volume with adequate inflow, outflow and biliary drainage [10]. There are no criteria for distinguishing between the patients for whom purely palliative treatment and those for whom potentially curative treatment is appropriate. Patients with LMCRC only have to be considered definitively unresectable after being treated with optimal systemic treatment over 2–4 months, so the possibility for resection is not missed in patients who a priori have a low chance of further R0 resection [11,12].

## 3. Conversion Chemotherapy to Improve Resectability

Conversion therapy could be defined as the use of chemotherapy to downstage the tumor, allowing to achieve a R0 resection in patients deemed unresectable because of high tumor burden. In this setting, highly active regimes with high response rates (RR) and minimum time to response are considered the best options, as a positive correlation has been shown between RR and resectability. However, this result has to be interpreted with caution, as regimes included in the study are different to those used nowadays [13].

Patients receiving conversion therapy should be reevaluated every two months [14] in order to avoid unnecessary toxicity for the liver, as oxaliplatin and irinotecan, two of the most used drugs in this setting, are associated with sinusoidal obstruction syndrome, non-cirrhotic portal hypertension and steatosis, respectively [15,16,17]. There are unresolved issues, such as the optimal time for reevaluation, the method of radiological evaluation, not only regarding the imaging test, but also the response criteria. Anti-angiogenic treatment is associated not only with changes in the size of lesions, but also morphological changes. Criteria based on these modifications have been described and have shown correlation with pathological response and survival [18]. As the efficacy of systemic drugs and agents are increasing, patients with LMCRC must receive 2–4 months of systemic treatment before considering them completely unresectable. At this time, the maximal tumor shrinkage is deemed to have occurred in most cases. No patient should lose the opportunity of an eventual resection although the a priori probability is deemed to be low [12].

Treatment options are chosen based on patient-related factors, the mutational profile of the tumor, and toxicity profiles of the chemotherapeutic drugs used. These drugs are given in doublets composed of a fluoropyrimidine with oxaliplatin, irinotecan or both. They are known as FOLFOX (5-FU + oxaliplatin), CAPOX (capecitabine + oxaliplatin), FOLFIRI (5-FU + irinotecan) or FOLFOXIRI (5-FU + oxaliplatin + irinotecan). All patients should have their RAS or BRAF mutation status determined [5,19]. Anti-EGFR therapy is not effective in mutant RAS patients, and the evidence increasingly suggests that BRAF V600E mutation is associated with poor response to panitumumab or cetuximab [20]. HER2 amplifications are also a matter of debate, as increasing evidence suggests that these patients are not expected to respond to anti-EGFR treatment [21]. NCCN guidelines recommend determination of HER2 amplification in all RAS and BRAF native patients, but ESMO guidelines do not recommend this determination outside clinical research. The role of HER2 in the conversion therapy setting is yet to be established [5,11,19]. High satellite instability and mismatch repair deficiency (MSI-H/dMMR) is predictive of response to immunotherapy but its role in conversion therapy is not defined. Sideness of the primary tumor is an important factor that must be taken into account as right-sided colon cancer is less responsive to anti-EGFR treatment. [22].

Table 2 summarizes the main trials of conversion chemotherapy, showing response rates and liver resection rates. Ye et al. showed that patients in either treatment arm undergoing hepatic resection had a significative longer median survival time than those who did not (46.4 versus 25.7 months for the cetuximab arm and 36.0 versus 19.6 months for the chemotherapy alone arm) [23]. A similar trend was shown in the PLANET-TTD trial, with a significative prolongation in OS in resected patients (52 months vs. 36 months) [24]. ESMO guidelines recommend for patients without RAS mutations and left-sided primary a chemotherapy doublet with an anti-EGFR antibody, as it is associated with a higher response rate. For right sided primary, a chemotherapy doublet or triplet (if appropriate) with bevacizumab is recommended. For RAS mutated tumors, a chemotherapy doublet or triplet is recommended. For BRAF mutated tumors, as BRAF is associated with poor prognosis, chemotherapy triplet plus bevacizumab is their recommendation [19]. The role of hepatic arterially directed therapies in conversion treatment are discussed below.

## 4. Local Ablative Therapies

There are many ablative percutaneous therapies, most of them consisting of direct administration of energy to destroy malignant tissue. A percutaneous probe should be placed in the metastatic lesion, all guided by image techniques as computer tomography (CT), magnetic resonance (MR) or ultrasound (US). The most important therapies are radiofrequency ablation (RFA), microwave ablation (MWA) and cryoablation [30]. Guidelines from ESMO support the role of a multidisciplinary team (MDT) in choosing the best treatment option from a “toolbox” of ablative therapies, taking into account some factors such as the size and number of metastases, rates of local control achieved and invasiveness of a particular technique, patient fragility, comorbidity and preferences and the local operator expertise regarding the use of a particular ablative treatment method. Discussion in a MDT is granted in complex cases but it is not mandatory in all cases [5,19]. On the other hand, the NCCN guidelines describe different ablative therapies, noting that the most extensive evidence published in the literature is about RFA. However, attention is paid to the growing evidence regarding other techniques, such as MWA, but no clear preference on one technique over the other is expressed. The SEOM guideline on LMCRC is in line with NCCN [11,12].

It is important to keep in mind that hepatic resection is only considered for *fit* patients (no medical contraindications to surgery), with favorable prognostic factors (number of lesions, presence or suspicion of extrahepatic disease, diverse scores) and with technically feasible surgery (diverse and evolving criteria; the most accepted criteria nowadays is whenever a macroscopic resection is feasible while maintaining sufficient liver remnant after the surgery) [5,19]. Results from surgery in these patients range from 20% to 58% of 5-year overall survival (OS) [31,32,33], but with 90-day mortality of 4% and a 40% of complication rate [34]. Ablative therapies were historically reserved for patients that were not candidates for surgery. Nowadays, it is very important to note that ablative therapies are considered potentially curative therapies in oligometastatic patients amenable to local ablation therapy with adequate margins, either alone or in combination with resection, as long as all visible disease is eradicable, even in patients considered candidates for surgery. This represents a significant change from the previous mindset [11]. In this line, the published literature suggests that ablation may provide acceptable oncologic outcomes for selected *fit* patients with small liver metastases that can be ablated with sufficient margins [35,36]. A panel of experts also noted the potential role and possible advantages of performing RF ablation during the interval between diagnosis and hepatic metastasectomy. This approach is known as the “test of time”. This would allow to cure a significant proportion of patients, to select patients for surgery for whom the ablation had failed, and to spare an unnecessary surgery for patients for whom new lesions appeared over the described interval and would be no longer candidates for surgery. This approach has been also proposed to progressing patients after an initial surgery as a modified test-of-time approach [37,38,39]. It is also very important to note that ablative therapies can be used in conjunction with surgery with curative intent to achieve complete local control with adequate surgical and ablation margins in patients in whom some lesions are resectable and others not. This is reflected in the three clinical guidelines previously described [5,11,12,19].

### 4.1. Radiofrequency Ablation

RFA consists in the placement of an electrode probe in the metastatic lesion for treatment, producing rapidly alternating electrical currents that produce ionic oscillations in water molecules, leading to the generation of frictional heat. Temperatures in the vicinity of the probe exceed 100 degrees Celsius, leading to instant cell death by thermocoagulation. Two types of RFA devices are usually used. Monopolar (MP) RFA and bipolar (BP) RFA employ a single antenna or two, respectively. Tissue destruction is greater near the probe, as the zone of active tissue heating is limited to a few millimeters near the probe. Blood flow in vessels > 3 mm can evacuate heat, limiting heat conduction in what is known as the “heat-sink” effect. BP-RFA is less affected by the heat-sink effect compared to MP-RFA [40]. Therefore, lesions located adjacent to noble structures, such as the central bile ducts, vessels > 3 mm and bowels are technically more complicated to correctly ablate. Tissue charring surrounding active elements of the probe also limits heat conduction and can contribute to a suboptimal procedure. Taking into account these technical considerations, the optimal lesions for this treatment are those smaller than 3 cm and not adjacent to noble structures. Incomplete ablation is more likely with increasing size, but electrodes 10 mm larger than the target tumor have been proved to successfully ablate larger diameter tumors [41]. Grounded pads placed in the lower extremities of the patient are required to protect from electric discharges. RFA can be conducted percutaneously or intraoperatively, previous to the procedure, concomitantly or after a planned or unplanned incomplete resection [42]. There are different approaches to electrode placement. The open technique is the most invasive approach and can be done concomitantly to liver resection or other simultaneous open procedures. The percutaneous approach is the least invasive procedure and can be performed without general anesthesia. It has is limitations, as lesions located in the hepatic surface or near other organs are difficult to treat with this approach. The laparoscopic approach tries to combine both to increase the proportion of treatable lesions and to minimize complications. This technique also allows for the identification of undetected liver tumors with the use of laparoscopic US [43]. This approach also allows to locate occult peritoneal or hepatic metastases not detected by conventional image techniques, enabling a more precise staging [37]. Local tumor recurrences in modern series appear to be low, and survival is similar as with other approaches [44,45]. Reductions in morbidity, cost and hospital stay as compared with open ablation have been reported [46]. Consensus documents recommend open approaches in patients undergoing resection with for unablatable disease, due to bulky disease or multiple clustered tumors in one area with ablation between one and four tumors in the remnant liver. The laparoscopic approach is recommended for tumors that are adherent to vulnerable structures. The percutaneous approach is preferred, as it is associated with less expense of hospital stay, morbidity and mortality [37]. Major complications are dependent on tumor size, electrode type, operator experience and number of ablation sessions. These complications are reported in 4–33% of patients. Major complications include intra-abdominal hemorrhage, tract seeding, liver abscess, intestinal perforation, portal thrombosis, biliary obstruction, pneumothorax, cholecystitis, cardiac arrest, and pulmonary embolism. Other, milder complications are ascites, biloma and celullites. One particular complication after thermal ablative therapies is post-ablation syndrome, which consists of low-grade fever, abdominal pain, malaise, myalgia, nausea, and vomiting. This complication is usually self-limiting in 7–10 days [6,47]. In observational and controlled studies, reported 5-year OS ranges from 27% to 50%, and 5-year recurrence free survival (RFS) ranges from 0% to 34%, with a local recurrence rate of 11% to 37% [33,48].

Prognostic tools have been developed to define risk populations of patients and to help to improve patient selection. The modified clinical risk score (CRS) for ablation, which includes five items (node-positive primary-tumor, disease-free interval from primary tumor to liver metastases < 12 months, number of tumors > 1, any hepatic tumor > 3 cm and pre-RFA CEA > 30 ng/mL) has shown to correlate with OS and local progression–free survival (LPFS) [36,39]. Other developed nomograms take into account other factors, such as the number of lesions, size of the biggest tumor, ablation margin < 10 mm and Ca 19.9 concentrations [49]. Interestingly, a nomogram was developed for unresectable patients receiving chemotherapy before RFA, which takes into account different validated prognostic factors, such as tumor size and number of lesions, but also response to chemotherapy, and it is designed for this specific clinical scenario, unlike other nomograms and scores [50]. The most important prognostic factor is the complete coverage of the targeted tumor by the ablation volume, with a sufficient ablation margin. Margins narrower than 5–10 mm are considered to be a poor prognostic factor for OS and LPFS [36,51,52,53,54,55,56]. Therefore, assessing correctly the margins achieved with the procedure is an aspect of capital importance. The effectiveness of the treatment is usually assessed with post-procedural imaging (US, CT, MR). The conventional approach consists in side-by-side juxtaposition of pre- and post-interventional CT scans and comparison by visual inspection, based on 2D images. Other different approaches have been proposed in order to improve outcomes and evaluation of the procedure. These approaches include the use of software to assess differences in size and attenuation of the lesions [57] or 3D software reconstructions of the ablated lesion to provide a quantifiable volumetric assessment of ablation completeness. This novel approach could challenge the traditional considered margins. A study has shown that ablations with a 100% circumscribed 3D safety margin of 3 mm or ablations with at least a 90% circumscribed 3D safety margin of 6 mm can be considered successful at the time of the intervention [58,59]. Other important prognostic factors are tumors > 3 cm, multiple metastases, old age, node-positive primary, metachronous metastases [60,61,62], RAS mutations [51,56], right-sided colon cancer [63], prior hepatectomy [64], negative Ki-67 and positive caspase-3 adhered cells to the electrode [65] and absence of viable cells in a biopsy of the ablated lesions with a marginal biopsy showing a minimal margin of > 5 mm after the RFA procedure [66].

Multiple studies, systematic reviews and meta-analysis have been conducted for patients with LMCRC. The EORTC-CLOCC was a phase II randomized trial that compared systemic chemotherapy with FOLFOX (and from October 2005 in combination with Bevacizumab) with or without RFA in 119 patients diagnosed with unresectable LMCRC. A total of 25% of patients in the combination arm had solitary metastases, compared to 11.9% in the chemotherapy only group. There was a significant difference between arms in OS: 3-, 5- and 8-year OS were 56.9%, 43.1% and 35.9% respectively, in the combination arm and 55.2%, 30.3% and 8.9% respectively, in the chemotherapy only group (hazard ratio (HR) = 0.58, 95% confidence interval (CI) = 0.38 to 0.88, *p* = 0.01). However, 45.9% of patients in the RFA arm required resection in combination with ablation to obtain a complete tumor treatment, which may have cofounded the results. Median PFS was improved from 9.9 months (95% CI = 9.1 to 12.9 months) to 16.8 months (95% CI = 11.0 to 21.9 months), similar to that described in OS (HR = 0.57, 95% CI = 0.38 to 0.85, *p* = 0.00). There were no differences in toxicity from the treatment [67]. Another phase II trial, the ARF2003, included 52 unresectable patients with LMCRC. Patients were treated with RFA (combined or not with surgery) 28 days after inclusion, and the primary endpoint of the trial was complete hepatic response at 3 months, with survival and safety as secondary endpoints. Neoadjuvant chemotherapy was allowed, and all patients received it before the procedure. Complete hepatic response at 3 months was 75%. The 1- and 5-year OS were 94% and 43%, respectively. LPFS at 1 and 3 years were 46% and 19% [68]

Regarding the comparison between RFA and surgery, RFA shows higher recurrence rate and poorer long-term OS, PFS and disease-free survival (DFS) compared to surgery, even in solitary metastases [60,69,70]. Thirty-day mortality after the procedure is similar between interventions but complications are more frequent in patients undergoing surgery [60,69,70]. For patients treated with the combination of RFA and surgery (for unresectable tumors), no significant differences (compared to surgery for resectable tumors) were noted in regards to OS and DFS, although worse local progression free survival (LPFS) was shown [69]. However, when similar ablation and resection margins can be achieved, reports have shown no difference between these techniques [71]. A study comparing outcomes from patients treated with RFA (±resection) from the CLOCC trial and only resection (from EPOC trial, a study of perioperative chemotherapy for LMCRC) was published. Although patients from CLOCC trial had a more advanced disease status compared with the resectable patients of the EPOC trial, local recurrence rates on a lesion basis were 6% and 5.5%, respectively. The 3 y OS rates were also similar (88% vs. 90%) [72]. The results from the COLLISION trial, a phase III trial currently recruiting, will compare surgical resection to RFA in CRC patients with lesions < 3 cm and are eagerly awaited, as they could answer whether RFA is non inferior in this population [34].

In recurrent colorectal liver metastases, the evidence is conflicting. Retrospective evidence by Dupré et al. suggest that surgery is associated with similar OS as RFA (median OS was not significantly different between the two groups, at 33.3 (95% CI 28–54.7) months. One, three- and four-year OS were, respectively, 97%, 30.4% and 30.4% in the RFA group and 92.9%, 52.2% and 43.5% in the surgery group). PFS was significantly worse in the RFA group (4.3 months vs. 10.2 months) along with LPFS (5.4 months vs. 11.8 months) [73]. In one single-center retrospective study, RFA was chosen as the first-line treatment in recurrent colorectal liver metastases. Patients not amenable to RFA were offered surgery instead. Local recurrence in the site of the procedure was different between groups, favoring surgery (32% in the RFA group and 4% in the surgery group, *p* < 0.001. This trend was also seen in time to progression. It was significantly shorter in patients primarily treated with RFA (203 vs. 416 days; *p* = 0.017) However, no significant differences between RFA and surgery were noted in appearance of new metastases in the liver (50% vs. 34%) or extrahepatic (32% vs. 37%). Of the liver-recurrent patients, 50% in the RFA group and 27% were candidates for reintervention (RFA or surgery). No differences in OS were noted between groups (3 y OS 67% vs. 60% *p* = 0.930) [74]. Prospective evidence by Dijkstra et al., in contrast, found no difference between LPFS, distant-PFS or OS between RFA and surgery in patients with mostly single metastases (64.5% of patients) and with small lesions < 3 cm (84% of patients). There were no difference in complications but significantly smaller hospital stay in patients treated with RFA [75].

### 4.2. Microwave Ablation

MWA generates frictional heat by creating electromagnetic radiation in the 900 to 2450 MHz range from an antenna placed in the lesion to treat. As this technique is associated with lesser heat loss in the surrounding tissue, the antenna provides a broader field of active heating, up to 2 cm from the antenna, in contrast to RFA. This allows to treat larger lesions by using multiple antennas. Other advantages of this technique are a shorter procedure time and the absence of grounding pads, and this may be safer for the patient [76]. Indications and contraindications to this procedure are similar to RFA as are the complication rate and severity [47].

Evidence is conflicting regarding the efficacy of MWA, with less data published. Median OS in case series range from 24 months to 40 months. The reported 3-, 4- and 5-year OS varies between 35% and 79%, 35% and 58%, and 17% and 32.9%, respectively [69,77]. Factors associated with worse OS and local control are tumor size (> 3 cm), tumor location (high-risk locations, such as near to a large vascular structure or diaphragm), number of lesions, elevated CEA levels prior to the procedure and the absence of response to chemotherapy before MWA [77,78]. One nomogram described previously also included patients treated with MWA and therefore, can be used in this population [49].

No trial has compared MWA in combination with chemotherapy vs. chemotherapy alone, but indirect observational evidence suggests greater survival with the combined approach [79]. One randomized clinical trial compared MWA to surgery in 30 patients with multiple (< 10), smaller than 8 cm, resectable liver metastases. There were no differences in median OS (25 vs. 27 months) or median DFS (13.3 vs. 11.3 months), with fewer post-procedure complications [80]. This study has its limitations, as there is an absence of *intention-to-treat* analyses with 25% of the randomized patients not included in the final analyses. A retrospective observational study comparing MWA to surgery found no significant differences in OS and DFS, and a 2018 systematic review found similar oncologic outcomes to resection [69,81]. A combined approach with MWA + surgery in patients in whom surgery is considered insufficient to achieve free margins vs. surgery alone in patients that are candidates for surgery showed no significant differences in recurrence-free survival (RFS), OS and DFS between these groups, with 19% of postoperative complications in the combination arm [82].

Pooled analyses and meta-analyses suggest no significant difference between RFA and MWA in terms of complications, but some suggest benefit in terms of the lower local tumor progression (LTP) rate and improved DFS and OS [70,83,84,85,86]. In a retrospective review of 110 patients, authors found no evidence of differences in local tumor control between these techniques when stratified by margins, stressing the importance of adequate margins (superior to 5–10 mm). However, importantly, unlike RFA, the efficacy of MWA ablation was not affected by perivascular tumors [55]. In comparison with laparoscopic RFA, laparoscopic MWA appears to have better local control but without significant differences in OS or DFS [84]. However, due to the limitations of the studies analyzed, randomized controlled trials are necessary to compare these two techniques. Ablation of resectable lesions with MWA will be allowed in the COLLISION trial, described before. This will provide valuable information about the efficacy of MWA in resectable patients in direct comparison with surgery [34]. MWA has also shown to be the most cost-effective strategy, compared to RFA and surgery, with the lowest overall cost and the highest cumulative cost-effectiveness, although in the resectable scenario [87].

Table 3 shows representative retrospective studies of RFA and MWA.

### 4.3. Cryoablation

Cryoablation, in contrast with the techniques described above, consists in the infusion of argon within a lesion through a percutaneous probe, thus dropping the lesion tissue temperature to −40 degrees Celsius. Tissue necrosis occurs from −20 degrees Celsius, with a direct effect on the neoplastic cells by causing membrane damage and protein denaturalization, and with an indirect effect caused by the vasoconstriction of blood vessels and hypoxic injury. This procedure can be performed percutaneously, intraoperative or in conjunction to resection to achieve free margins, in what is known as edge cryotherapy [92]. The size of the treatment field depends on the configuration of the probe. There are some advantages to this procedure, as it causes lesser pain to the patient, and the treatment field can be delimited more easily with a post-procedure CT or real-time intraoperative US. However, there are important disadvantages, as the tissue adjacent to great vessels may not fall to a sufficient temperature to cause cell death, similar to a “reverse” heat-sink effect [93]. The lack of a coagulative effect on the surrounding tissue can also contribute to potential bleeding complications. Another important concern with the use of cryotherapy is the cryoshock phenomenon. The absence of a precise definition makes it difficult to establish an approximate incidence. Patients suffering from cryoshock have disseminated intravascular coagulation and multi-organ failure, similar to septic shock but without any microbiological isolates. A large multicenter survey estimated that it was responsible for 18% of peri-procedural deaths [94], but more recent series report a very low incidence [92,95,96]. The high rate of complications of earlier series and the fear of cryoshock led to this technique being displaced in favor of other ablative techniques [97].

A Cochrane systematic review was published to assess the role of cryotherapy for the treatment of liver metastases. Only randomized trials were included in the analyses. After review of the literature, only one randomized clinical trial was found [98]. This trial randomized 123 consecutive patients (82 patients with LMCRC) to cryosurgery versus conventional surgery. A high risk of bias is present, as there is no information provided to assess the sequence generation or allocation concealment [99]. The cryosurgery consisted of diverse procedures, including cryoresection using a self-constructed cryogenic clamp to freeze the desired incision line, and cryoablation. Mortality at 3, 5 and 10 years was 40%, 56% and 81% in the cryotherapy group and 49%, 64% and 92% in the conventional surgery group, respectively. No significant differences were found. Reported adverse events were similar between groups (10% and 20%), except in the case of pain, which was worse in the cryotherapy group [98,99].

Case series report diverse 1-, 3- and 5-year OS, ranging between 46% and 92%, 8% and 60%, and 0% and 44%, with major complication rates ranging from 1.5% to 66% and minor complication rates between 20% and 55%. Edge cryotherapy case series report similar survival to R0 resection, but major complications compare unfavorably to major hepatectomy [95,96,97,100].

Table 4 shows the main randomized clinical trials regarding RFA, MWA and cryoablation.

### 4.4. Other Ablative Therapies

#### 4.4.1. Laser Interstitial Thermal Therapy (LITT)

LITT involves the placement of laser fibers (usually neodymium-doped yttrium aluminum garnet (Nd-YAG)) in the lesion to treat, inducing electromagnetic heating by the conversion of light into heat and causing coagulation necrosis [101]. These laser fibers can be introduced percutaneously or by endoscopic ultrasound [102]. Conventional fibers allow zones of 1–2 cm to be ablated with this technique, as the surrounding tissue absorbs light and limits heat transferring [103]. Newer interstitial fibers with diffusing tips can provide larger ablation areas up to 50 mm [104]. To ablate larger volumes, multiple fibers need to be placed, but caution is advised, as the carbonization of the skin is a potential complication of high power ablation treatments [105]. This technique also allows to monitor temperature maps on the tissue in real time with CT or MR, due to the lack of metal and the small diameter of the applicators [106]. General anesthesia is not needed. This technique is not feasible in patients with more than 5 lesions and lesions greater than 5 cm in diameter [107].

Literature reviews suggest a local progression rate of 5.2% to 10% at 6 months of follow-up [47]. In a cohort with long-term follow up, mOS from the time of first LITT was 25 months. mOS in the patients treated with curative intent was 29 months vs. 21 months in patients treated with palliative intent. Significant prognostic factors in multivariate Cox analyses were the number and diameter of the biggest metastases, as well as the primary lymph node stage. Patients with one metastasis showed a mOS of 30 months, while patients with ≥ 4 lesions had 18 months of mOS. In patients with tumors < 20 mm, mOS was 36 months, while patients with > 40 mm metastases had an mOS of 21 months. mPFS was 13 months with no differences regarding the intention of the treatment. A stepwise backward hazard ratio model revealed the number of metastases and their diameters as significant prognostic factors. In patients with lesions < 20 mm, mPFS was 21 months. In patients with metastases > 40 mm, mPFS dropped to 10 months. Similarly, patients with up to 1 metastases had a mPFS of 18 months, while patients with more than 4 metastases had a mPFS of 10 months [108]. The major complication rate ranged from 0.1% to 3.5% with pleural effusion, subcapsular hematomas, abscess, pneumothorax, pleuritis, intrahepatic hemorrhage and biloma as the main major complications described in the literature [47,109].

LITT allows the use of finer needles than RFA or MWA, so endoscopic ultrasound (EUS) guidance has drawn some attention as a novel technique that would allow to ablate lesions at the left or caudate lobe of the liver since these locations are frequently hard to ablate percutaneously. A phase I trial was conducted, which included 3 patients with LMCRC, with no major complications reported. At 3 months post-procedure, MR showed an absence of enhancing in the treated lesions. No long-term results were reported. This study is the first to describe the feasibility of this procedure in ablating LMCRC endoscopically [102].

#### 4.4.2. High-Intensity Focused Ultrasound (HIFU)

HIFU is based on the production of high-intensity ultrasound waves at a low frequency (0.8–1.6 MHz) by a transducer outside of the body. The US waves are focused into a small region (with a range from 1 × 1.5 mm to 10 × 16 mm). This raises the temperature of the affected tissue > 55 degrees Celsius, causing thermocoagulation necrosis. At higher acoustic intensities, the generation of oscillating gas bubbles that accumulate heat due to mechanical friction with US waves can also cause tissue damage by cavitation. Cavitation is caused by mixed thermic and mechanic tissue damage caused by collapsing gas bubbles [110].

The key advantage of this form of ablation is that it is performed noninvasively, minimizing the risk of bleeding or unintentional organ injury. Nonetheless, if the lesions to be treated are located at the hepatic dome near the diaphragm, an artificial pleural effusion must be created to improve the sonographic window [111]. Other authors describe the removal of the ribs to improve the sonographic window and diminish the osteonecrosis risk, altering the non-invasive nature of this procedure [112]. There have been attempts to adapt this technique to the intra-operative setting, without compromising asepsis of the procedure [113]. The main disadvantage of this procedure is that each application of HIFU can only encompass a small volume of tissue, so sequential treatments are needed to cover larger volumes of tissue, resulting in long ablation and anesthesia times [114]. The most frequent HIFU complications are skin burns on the application site and osteonecrosis of the bones along the US pathway. Other potential complications, although irregularly reported, are fever and pleural effusion [112].

There are not published phase III trials of this technique for the treatment of colorectal liver metastases, although there are positive phase III trials in the symptomatic treatment of painful bone metastases and for the treatment of hepatocellular carcinoma (HCC), compared to the use of trans-arterial chemoembolization only (TACE) [115,116]. There are retrospective data from a study which included 43 colorectal patients, not candidates for resection, for whom HIFU was selected as the ablation technique. There are no reported data about why HIFU was the technique chosen. In this cohort, no complete responses (CR) by RECIST 1.1 criteria were seen, but an ORR of 39.5% of colon and 64.5% of rectum patients was reported. mOS was 12 months for both, and 1-year OS was 66.6% and 86.67%, respectively. Adverse prognostic factors were ECOG ≥ 2, longest lesion diameter ≥ 5 cm, the presence of extrahepatic metastases and portal vein invasion [117]. Prospective data come from two small phase 1 trials. In the first one, 10 patients not candidates for surgery that specifically asked for HIFU treatment were treated. The rate of primary effectiveness (percentage of patients without progression of the treated lesions among the total) was 20%, with 100% of minor complications, particularly osteonecrosis of the rib, and 20% of major periprocedural complications, leading to the death of a patient. No other outcomes were reported [111]. Another phase I trial, designed specifically for patients not candidates to resection or RFA by technical criteria and without extrahepatic disease was recently published in which 13 patients were included. ORR by mRECIST was 100%, with 76.9% of CR. The locorregional failure rate, defined as the appearance of new lesions surrounding or in the same hepatic segment, was 61.5%. An additional HIFU treatment was feasible in all recurring patients, but the ORR was 89%. mPFS was 9 months, with a 2-year PFS of 16.7%. mOS was 25 months with a 2-year OS of 77.8%. A total of 84.6% of the patients received systemic therapy after HIPU, and chemotherapy could be resumed a week after the procedure. Adverse events (AE) occurred in 69.3% of patients with no grade 3–4 AEs. No osteonecrosis of the rib was noted [118].

#### 4.4.3. Irreversible Electroporation (IRE)

Irreversible electroporation (IRE) uses short, powerful electric fields (70–90 pulses of high energy 1000–2500 V/cm^2^ with 100 msec of duration) to create membrane pores in tumor cells, causing membrane disruption and leading to cell apoptosis, not coagulative necrosis as RFA or MWA [119,120]. Proteins are not damaged, so the acellular component of the stroma is not affected by this technique [120]. A critical advantage of this technique is the avoidance of the “heat sink” effect described for RFA and MWA, as the short duration of pulses allows the tissue to cool and avoid thermal effects [121], and cell death is not dependent on coagulative necrosis. Some experiments have showed that under certain circumstances, thermal coagulation on the tissue is present with IRE [122], but this temperature increase is not considered to be detrimental to the surrounding connective tissue at the recommended settings. Nonetheless, placement of the electrodes ≤ 2 mm to large vascular structures is not recommended, and, if inevitable, the electrode placed should be the positive one, as it is the colder one. Metallic objects, such as stents, should also be avoided because they can cause alterations in the electric field distribution and could compromise the effectiveness of the procedure [123].

This treatment is currently only indicated for tumors not amenable for surgical resection or thermal ablation. This technique is most effective for tumors ≤ 3 cm, with patients with more than four metastases being suboptimal candidates [124]. Its main application is in the treatment of central lesions with large vessels or bile ducts in proximity [125]. IRE can be performed during laparotomy or percutaneously, guided by CT or US. General anesthesia is needed, as a muscular relaxation on the patient is mandatory to avoid uncontrolled muscle contractions causes by the electric fields. Cardiac monitoring is also required, as ventricular arrythmias are a serious potential complication of this technique. It is important to note that modern devices are synchronized to the electrocardiogram (ECG) of the patient to deliver pulses in the refractory period of the cardiac contraction to avoid this complication. Other potential complications are hepatic abscesses, pneumothorax, bleeding, portal vein thrombosis and bile duct leakage or occlusion [124,126]. Needle tract seeding following IRE was also described [127]. In a systematic review of retrospective studies, the overall complication rate (OCR) was 16% with no major complications reported [124]. In a single-institution series of 85 IRE ablation procedures, major complications occurred in 7.1% of patients, while minor complications occurred in 18.8% [128]. In another study comparing RFA and IRE for the treatment of HCC, no differences were noted regarding the post-procedure complication rate, post-procedure hospital stay, or frequency of post-procedure ICU stay or length. The intervention time was longer in the IRE patients [129].

In regard to the treatment of liver metastases of colorectal origin, a phase II trial was recently published [130]. The COLDFIRE-II is a single-arm, multicenter study that included 51 patients with liver-only metastatic colorectal cancer, with at least one ^18^F-FDG PET-avid lesion measuring ≤ 5 cm considered anatomically unsuitable for resection and in which thermal ablation was contraindicated, due to tumoral vicinity to a major bile duct, abutment of a single remaining major portal vein or hepatic vein and unreconstructable invasion of the free wall of the inferior vena cava. Patients did not receive adjuvant systemic therapy following IRE, but neoadjuvant treatment was allowed. Multimodal treatment for other liver tumors as concurrently thermal ablation or surgery was also allowed. The study met its primary endpoint with a 1-year local PFS of 68%. Median distant PFS was 5.3 months, the liver being the most frequent site of first recurrence. Median extrahepatic PFS was 12.5 months. mOS from first IRE was 2.7 years. Eight patients underwent repeat treatment with IRE. Local tumor control in the repeated procedures was achieved in 74%. The overall complication rate was 40%. The most frequent grade 3–4 AEs were de novo biliary obstruction, periprocedural arrythmias and portal vein thrombosis. A treatment-related death due to an infected biloma was reported [130]. Systematic reviews of retrospective series report a primary efficacy of the procedure within a range of 67–100% and secondary efficacy within 55–93% [124]. In a study reporting long-term follow up of patients treated with IRE, complete ablation after first IRE was achieved in 66.7% of patients, and 95.8% after reintervention. No differences in OS were noted regarding tumor size (small vs. ≥2 cm). The OS of the entire cohort was 26.5 months, with 1-, 3-, and 5-year OS of 79.1%, 25% and 8.3%, respectively [131].

#### 4.4.4. Stereotactic Body Radiotherapy (SBRT)

SBRT is defined as an external beam radiotherapy used to deliver a high dose of radiation very precisely to an extracranial target within the body, as a single dose or a small number of fractions [132]. It represents a non-thermal and non-invasive ablation technique. In order to achieve high-precision delivery, motion management techniques are needed in order to avoid unnecessary radiation of the surrounding tissue. The new approach of MR-guided adaptative therapy is a novel technique for delivering treatment, coupling MR-guided acquisition of images before, during and after the delivery of treatment. This allows for better monitoring for in-treatment changes in position of the lesion to treat. Additionally, it allows for a better delimitation of tumors in the liver [133]. For liver metastases, fiducial placement is usually used to improve correct treatment delivery. In MR-guided SBRT, no fiducials are needed, avoiding an unnecessary invasive procedure [133]. Apart from the treated lesion, a margin of 5 mm of normal tissue is usually added. Different schemes and fractions are used, but typical dose schedules range from 3 to 8 fractions delivering 45–60 Gy for liver metastases [134]. CRC is considered radioresistant, but this resistance can be overcome by the high doses achieved with this technique [135].

Eligible lesions for SBRT must be below 5 cm in maximum diameter. SBRT, unlike thermal ablation therapies (specially RFA), can be used in lesions adjacent to great vessels, as no heat-sink effect is present, and lesions near other organs (subcapsular, periampullary) where colocation of an electrode is risky. However, caution must be taken in the planification of the treatment, as other tissues with different radiation sensitivity can be harmed during the delivery of the treatment, such as the GI tract [136]. Other reviewed techniques could be useful in this context (i.e., hydrodissection during thermal ablation, IRE, and radiation segmentectomy). Guidelines from ESMO and NCCN note that, as other ablative therapies, SBRT could represent a potentially curative treatment, alone or in combination with surgery to achieve no evidence of disease and represent an important option for patients not amenable to surgery or other forms of ablation [5,11,19].

SBRT can achieve a local control rate up to 90% at 24 months depending on size [137,138]. Results from two phase II trials were recently published. In an international trial, 99 patients (18.2% of patients with CRC) with a controlled primary tumor and up to 5 metastatic lesions were randomized to systemic palliative therapy versus standard of care and SBRT of all metastatic lesions. This study showed benefit in the SBRT arm in terms of PFS and OS (6 months vs. 12 months and 28 months vs. 41 months, respectively). Adverse events grade ≥ 2 were more common in the SBRT arm [139]. However, several limitations of this trial must be noted, as compared groups are very heterogeneous in primary histologies and metastases in different sites. No information is provided regarding the use and timing of systemic therapies. These limitations make the trial results difficult to interpret and extrapolate, as was noted by a multidisciplinary expert comment [140]. In the other published phase II trial, 42 patients not amenable for surgery or RFA with liver-limited disease, and with no more than 3 lesions with a diameter < 6 cm, were included. Complete response was achieved in 22 (43%) lesions, partial response in 17 (32%), and stable disease in 9 (17%). mPFS was 12 months and mOS was 29 months. No grade ≥ 3 toxicity was found, but G2 hepatic toxicity was 78%. Univariate analyses showed that OS was worse in lesions > 3 cm [141].

Data from a systematic review found that local control and PFS can be achieved, even in pretreated patients, and data from retrospective databases confirmed the benefit that can be achieved in patients for whom control of all metastatic disease can be achieved. However, relapses can be present in up to 31% of treated patients. Local control was 67% at one year, while the pooled two-year LC was 59.3%. Pooled one- and two-year OS were 67.18% and 56.5%, respectively. Median PFS and OS were 11.5 and 31.5 months. Correlation analyses found a significant correlation between dose and local control and OS, but with a poor linear correlation [9,142]. One retrospective study compared outcomes between patients who received MWA vs. patients who received SBRT in a tertiary care center. This study showed better freedom from local progression (FFLP) in patients receiving SBRT (1-year FFLP 91% vs. 84%), especially in those with lesions > 3 cm [143]. Another study comparing MWA to SBRT in patients included in AmCORE registry showed conflicting results. In this study, SBRT was associated with worse OS, even in patients with lesions < 3 cm, (mOS median OS 53.0 months vs. 27.4 months) and worse LTP (29% vs. 8.9%), compared to thermal ablation. Patients treated with SBRT were older, with larger lesions and more extrahepatic disease [144]. Neither of these two studies explore the role of ablation margins and do not stratify results by this important prognostic factor. Other retrospective databases from international consortiums underscore the role of registries in standardizing the treatment of our patients, as the optimal regime has to be defined. These retrospective databases confirm the heterogeneity of the regimes used and suggest that the size of the treated lesions and dose administered could be considered prognostic factors [145,146].

## 5. Hepatic Arterially Directed Therapies

Locoregional therapies administered through the hepatic artery for the treatment of LMCRC include chemoinfusion, chemoembolization and radioembolization. Hepatic malignancies receive 80% of their blood supply from the hepatic artery in contrast to the normal liver, which receives 80% of its blood supply from the portal vein and 20% from the hepatic artery [147]. Hepatic arterially directed therapies through the hepatic artery target the cells of LMCRC with relative sparing of normal liver parenchyma.

### 5.1. Chemoinfusion

Chemoinfusion (CI) or hepatic artery infusion is the delivery of chemotherapy (CT) directly into the hepatic artery that results in high exposure of the liver to the chemotherapeutic drug. CT can be delivered through catheters placed percutaneously into the hepatic artery or through pumps and catheters that are surgically implanted [148,149]. The most commonly used drug is floxuridine (FUDR), an antimetabolite of fluorouracil, because it has a high first pass clearance by the liver: it increases hepatic exposure and decreases systemic exposure. Irinotecan and oxaliplatin have been also used [150,151]. CI may present several adverse events, such as catheter malfunction, arterial occlusion, hepatic toxicity or biliary sclerosis [152,153].

CI was compared to systemic CT for first-line treatment for unresectable LMCRC. Several prospective clinical trials published in the late 1980s to early 1990s comparing CI with QT demonstrated superior response rates of CI therapy but did not show consistent improvements in OS [154,155,156]. Two meta-analyses confirmed these results [157,158].

The improvements in the CI technique of pump placement, the methods of optimizing pump CT and the development of new therapies generated a new interest in CI, so new studies have explored the use of CI in conjunction with QT. A randomized trial compared treatment of LMCRC using CI FUDR, LV and dexamethasone to systemic CT (5-FU and LV). mOS was 24.4 months for CI vs. 20 months for CT. The time to hepatic progression was 9.8 months for CI vs. 7.3 months for CT. However, the time to extrahepatic progression was 7.7 months for CI vs. 13.8 months with QT [159]. A study of 153 patients randomized to receive CI FUDR alone or CI FUDR and systemic 5-FU as first-line therapy demonstrated no difference in ORR (52.7% vs. 50.6%) and OS (18.0 vs. 19.1 months) [160].

Most institutions have already abandoned the use of CI alone, so diverse studies have tested the efficacy of CI with a combination of oxaliplatin and irinotecan. A phase I study with 49 patients with unresectable LMCRC treated with CI FUDR and dexamethasone and CT (oxaliplatin and irinotecan) demonstrated a high ORR (92%) and a conversion to resection (47%), with a mOS of 51 months [161]. A total of 87 patients received CI oxaliplatin with 5-FU and LV as second-line treatment of LMCRC, and 24% of patients underwent resection, with 5-year OS of 56% [162]. A total of 28 patients with LMCRC received CI oxaliplatin followed by intravenous CT (5-FU and LV). The ORR was 64%, and mOS was 27 months [163]. Recent studies demonstrated high response and conversion to resection rates, using combinations of modern systemic agents (Table 5) [164,165,166,167].

CI as adjuvant treatment has demonstrated mixed results. Three studies showed increase in PFS and OS [168,169,170], but another study did not show any survival benefit [171]. In 2014, a Cochrane review of seven available randomized adjuvant CI studies showed no increase in OS [172].

A study followed 287 patients that underwent adjuvant CI with FUDR and systemic QT after resection of LMCRC, from 1991 onward. The patients were divided into two groups: those treated before 2003 and after. Results of this study showed greater 5- and 10-year OS in those treated after 2003, compared to before 78% vs. 56%, and 61% vs. 40%, respectively. These differences were thought to be secondary to new targeted therapies, more aggressive surgical treatment of recurrences, and better imaging [173].

Subsequent studies considered whether or not CI therapy was given with perioperative modern systemic therapy as a subgroup analysis to further delineate the contribution of modern QT [174]. A study of 21-year analysis of perioperative CI included 2368 patients with resected LMCRC from 1992 to 2012. The results showed prolonged 5-year OS for patients receiving CI therapy, compared to those treated without CI (52.9% vs. 37.9%) [175]. For those that received preoperative modern systemic QT, the median OS in the CI arm and the no-CI arm were 77 and 45 months, respectively. For those that did not receive preoperative modern systemic QT, mOS rates in the QT arm and the no QT arm were 55 and 43 months, respectively [176]. Despite the increased effectiveness of modern systemic QT, there is still a benefit in OS with the addition of CI therapy to systemic therapy in the perioperative setting.

The place of CI in the therapeutic armamentarium for mCRC is not clear. NCCN guidelines consider CI as an option of treatment in resectable and unresectable LMCRC, but it should be considered selectively and only at institutions with experience in this procedure. It is an acceptable first-line option in the United States for unresectable LMCRC and a treatment option in the adjuvant treatment, but it remains infrequently used [177,178]. However, ESMO guidelines do not recommend CI [5]. Spanish guidelines regarding the management of LMCRC do not mention CI [12].

### 5.2. Chemoembolization

Transarterial chemoembolization (TACE) adds arterial obstruction to the delivery of a chemotherapeutic drug. The most commonly employed chemoembolization protocol combines the delivery of chemotherapeutic drugs emulsified in ethiodized oil with particulate embolization. The ethiodized oil/chemotherapeutic mixture lodges distally within the hepatic arterioles and portal venules, trapping the chemotherapeutic drug in the tumor microvasculature. The embolization following the delivery of the ethiodized oil/chemotherapeutic mixture leads to stasis and increased contact time within the tumor, which increases local drug delivery, reducing systemic exposure [179,180]. This combined treatment results in the retention of the chemotherapeutic drug in the tumor cells for several weeks, while they clear from normal hepatocytes within seven days [181].

Chemotherapeutic agents, such as doxorubicin or irinotecan, are ionically bound to particles of various sizes. These include hydrogels, microspheres and polymer implants. The most extensively studied are non-biodegradable polyvinyl alcohol (PVA) microspheres (beads) (DC-Bead, Biocompatibles, West Conshohocken, PA, U.S.A.). The beads are joined sulfonic acid containing a moiety producing a charge that permits the interaction and binding with oppositely charged chemotherapeutic agents [182]. Beads of varying diameter (70–900 microns) are loaded with chemotherapeutic agents, and they are delivered in a lobar, segmental or superselective arterial distribution.

Drugs eluting beads loaded with irinotecan (DEBIRI) is a useful alternative for TACE in LMCRC [183,184]. DEBIRI loco-regional activity and pharmacologic profile differs from doxorubicin. Therefore, irinotecan is preferred in LMCRC, and doxorubicin is mostly used in HCC. [185]. The irinotecan is released from DC-Beads inside LMCRC, and DC-Beads occlude liver arterial vessels. However, the ischemic effect alone is not enough to justify the extent of the tumor response observed, so the irinotecan effect is required. The use of DC-Beads alone results in a lower tumor response than what is observed with different doses of DEBIRI, confirming the importance of both embolization and drug activity [186]. Another result of embolization-induced ischemia is the decrease in liver pH that activates irinotecan.

The most common complication following TACE is postembolization syndrome that is caused by the release of cytokines and subsequent inflammation by tissue ischemia [187]. This syndrome appears in 30–80% of patients, with right upper quadrant pain, nausea, vomiting and fever. Additional complications include liver failure, hepatic abscess formation, biloma, biliary ischemia or ductal injury [188].

A study with 121 patients with LMCRC treated with TACE (mitomycin C, doxorubicin and cisplatin with ethiozied oil) described that 41% and 57% of patients had stable disease and disease progression, respectively [189]. Median time to liver progression was 5 months and mOS following the first TACE was 9 months. These results are similar to other older series, that reported mOS of 8–14 months from the time of first TACE [190,191].

Table 6 shows the most important results of studies that analyzed the paper of chemoembolization as the first-line treatment of LMCRC. One study evaluated the role of the addition of conventional TACE (cisplatin + 5-FU) to systemic QT in first-line treatment of unresectable LMCRC [192]. It was a retrospective 4-arm study of 154 patients with KRAS wild-type LMCRC comparing QT (FOLFOX/FOLFIRI) +/− cetuximab vs. QT (FOLFOX/FOLFIRI) +/− cetuximab plus TACE. mOS was different between the study arms, as 5-years PFS. A phase 2 trial tried to evaluate in 70 patients the benefit of the addition of DEBIRI to systemic therapy in the first-line treatment of unresectable LMCRC: FOLFOX with or without bevacizumab vs. FOLFOX with or without bevacizumab plus DEBIRI [193]. However, the patients in the two arms were not similar at the baseline evaluation (patients from DEBIRI intervention arm had worse performance status and more extrahepatic disease). There were no differences in response to treatment between the study arms at 2, 4 and 6 months using RECIST 1.1, but there was a significantly better response at 2 months in the DEBIRI arm (98% vs. 82%).

Several studies analyzed the benefit of DEBIRI as second or later line treatment in patients with unresectable LMCRC (Table 7). A phase 2 trial that studied DEBIRI in 82 patients reported that mOS was 25 months, mPFS was 8 months and ORR was 78% 3 months after DEBIRI [194]. Another phase 2 study analyzed 40 patients treated with DEBIRI plus capecitabine. mOS was 8 months and PFS was 4 months [195]. A phase 3 trial with 74 patients compared DEBIRI to FOLFIRI. OS and PFS was longer in the DEBIRI arm (22 months vs. 15 months, 7 months vs. 4 months, respectively). Similarly, ORR and time to hepatic progression were better in the DEBIRI arm (68.6% vs. 20%, 7 months vs. 4 months). An evaluation of KRAS demonstrated that within the DEBIRI treatment arm, those with wild-type KRAS had better OS than those with mutated KRAS (26 months vs. 14 months) [196].

These studies suggest a benefit in terms of OS and PFS in the second-line setting, although the strength of evidence is low, owing to the small sample size. Treatment with DEBIRI may provide benefits to patients with liver predominant metastases whose disease has progressed during first-line systemic therapy. NCCN guidelines do not realize specific recommendations for DEBIRI. The TACE treatment is not described in the ESMO guidelines [5]. Spanish guidelines consider that DEBIRI is indicated as a third-line treatment when QT has failed in some non-resectable LMCRC and could provide an opportunity for some patients who need downstaging prior to surgery [12].

### 5.3. Radioembolization

Radioembolization (RE) or selectivity internal radiotherapy uses Yttrium-90. Yttrium-90 is a radioactive beta emitter (maximum energy 2.27 MeV, mean range of 2.5 mm in liver tissue and half-life of 64.8 h) embedded in resin (SIRSpheres, SIRTEX) or glass microspheres (Theraspheres, MDS Nordion) for delivery of high dose radiation to the tumor with reduced radiation exposure to the normal liver parenchyma [197,198].

The RE procedure consists of a catheterization of the hepatic artery through a femoral access to deliver Yttrium-90 microspheres to the hepatic parenchyma. These particles occlude the smallest capillaries, leaving the majority of the microspheres within the tumor, whereby they emit radiation therapy. The particles are radioactive for a period of 14 days but most of the radiation is delivered over five days. SIRSpheres and Theraspheres are used interchangeably, but SIRSpheres (20 to 40 μm) are approved for LMCRC and Theraspheres for hepatocellular carcinoma [199].

The RE process is realized in at least two parts. In the first session, a mapping portion is made. A Technetium-99 macro-aggregates albumin SPECT (single photon emission computed tomography) during which particles mimicking the Ytrrium-90 microspheres determine the percentage of lung shunting, which may occur with the RE procedure. If greater than 20% lung shunting occurs, the patient is not eligible for RE. The Yttrium-90 dose may be modified based on the percentage of lung shunting. Additionally, during this procedure, occlusion of the gastroduodenal artery may be realized to prevent retrograde flow of the microspheres, which can make gastric and duodenal ulcers [200]. The second procedure is the administration of the Yttrium-90 microspheres, which may be performed in a whole liver approach or sequential lobar treatments. The advantage of sequential treatments is to observe the effect of first RE and to assure that a sufficient contralateral liver reserve exists.

One important adverse event of RE is radiation-induced liver disease (RILD). The Yttrium-90 microspheres preferentially flow to the tumors due to the hepatic artery–dominant blood supply for about 80% of liver tumors. The distribution of microspheres was confirmed in pathological studies [198]. RILD is a constellation of icteric ascites, hepatomegaly, and mild elevation to transaminases relative to bilirubin, which is markedly elevated. Patients who have been treated with QT prior to RE have higher risk for RILD [201]. Other risks of RE are constitutional symptoms, abdominal pain or gastric/duodenal ulcer.

There are not comparative studies studying if RE with Yttrium-90 is useful in patients with resectable LMCRC [202]. Several clinical trials have showed potential benefit for combining Yttrium-90 with intravenous fluoropyrimidine-based QT in patients with LMCRC [203,204]. RE with Ytrium-90 significantly improved PFS (4.5 months vs. 2.1 months, *p* = 0.03) and liver PFS (5.5 months vs. 2.1 months) but differences of OS were not significant (7.3 months and 10.0 months, *p* = 0.80) [204]. The efficacy of combined therapy with Ytrrium-90 plus systemic QT over systemic QT alone as first-line treatment in patients with unresectable LMCRC was studied in three parallel phase III trials (SIRFLOX, FOXFIRE and FOXFIRE-Global) [205,206,207]. The three trials had very similar eligibility criteria and had a combined enrollment of 1103 patients who were randomly assigned to FOLFOX (+/− targeted therapy) or FOLFOX (+/− targeted therapy) plus Ytrrium-90 concurrent with cycle 1 or 2.

Only the results of SIRFLOX study were fully published, and the others were included in abstract presentations. There is a pooled analysis of these three clinical trials in different publications [208,209,210,211,212]. A total of 549 patients were treated with QT alone and 554 were treated with QT plus Yttrium-90, at a median follow-up of 43 months. There was a higher ORR with combined therapy (72% vs. 63%) but there were no differences to mOS (22.6 months vs. 23.3 months) or mPFS (11 months vs. 10.3 months) [208]. There was significantly longer liver disease control in the Y90 group by 8 months (20 months for Y90 vs. 12 months for QT) in the SIRFLOX trial. Table 8 shows the most relevant results. Combined therapy had more grade 3 or 4 adverse events, especially hematologic toxicity. There were 11 treatment-related deaths: 8 were in the QT plus Ytrrium-90 group and 3 were attributed to RILD.

There are results of subgroup analyses of FOXFIRE, SIRFLOX and FOXFIRE-global. There were no OS differences between the treatment arms based on the KRAS mutation status [210]. OS was significantly greater in the QT plus Yttrium-90 in those with right-sided tumors (22 months vs. 26 months), but not left-sided tumors (24.6 months vs. 26.6 months) [211]. Similar results with respect to tumor localization were demonstrated when only the SIRFLOX and FOXFIRE-Global trials were combined [212].

Patients with LMCRC who have failed multiple systemic QT regimens may benefit from RE with Yttrium-90 [213,214,215,216,217,218,219,220]. A systematic review of 20 studies comprising 979 patients found that rates of complete radiologic response, partial response, and stable disease were 0% (0–6), 31% (0–73), and 40.5% (17–78), respectively [221]. The median time to intrahepatic disease progression was 9 months, mPFS was 4.9 months, and the mOS was 12 months. Acute toxicity developed in 11 to 100 percent (median 41 percent), most of which was mild. Only one small phase 3 clinical trial evaluated systemic QT alone (5-FU) and systemic QT (5-FU) plus RE with Ytrrium-90 [204]. There were differences in mPFS (2.1 months vs. 5.5 months), but mOS was similar in the two arms (7.3 months vs. 10 months). Recently, it has become known the results of the EPOCH trial [222], which was a phase 3 trial that demonstrated the effectiveness of the addition of RE to second-line chemotherapy. mPFS was 7.2 months in the control group, mPFS was 8.0 months in the RE group and the HR of PFS was 0.69 (95% CI, 0.54 to 0.88; 1-sided *p* = 0.0013). So it reflects a 31% protection against progression in Y90 group. The median hepatic progression-free survival was 9.1 months in the RE group and 7.2 months in the control groups, and the HR was 0.59 (95% CI, 0.46 to 0.77; 1-sided *p* < 0.0001. mOS was 15.2 months in the RE group and 14.3 months in the control group. A significant benefit with the addition of RE was observed for patients with tumors with a KRAS mutation, left-side primary tumors, a tumor burden of 10–25%, three or fewer lesions, the addition of a biologic agent and no detectable extrahepatic lesions.

It was investigated if there are several factors that could improve OS and liver PFS in patients treated with Y90. Kurilova et al. proposed a normogram that can predict OS in patients treated with Y90 and pretreated with other treatments. This normogram includes six pre-Y90 treatment parameters: number of extrahepatic disease sites, carcinoembryonic antigen (CEA), albumin, alanine aminotransferase (ALT) level, tumor differentiation level and SUVmax of the two largest tumor diameters. Baseline SUVmax was the single significant predictor of liver PFS [223].

It is not easy to know how to incorporate RE with Yttrium-90 in the treatment of patients with LMCRC. The Radioembolization Brachytherapy Oncology Consortium (RBOC) suggests that RE with Yttrium-90 be limited to patients with unresectable LMCRC–dominant tumor burden and a life expectancy over 3 months [213]. RBOC defends that an absolute contraindication to Yttrium-90 is a pretreatment Technetium-99 macro-aggregated albumin SPECT with ≥30 Gy radiation exposure to the lung or flow to the gastrointestinal tract that cannot be corrected by catheter techniques. Other relative contraindications would be a limited hepatic reserve, irreversibly elevated serum bilirubin levels, a compromised portal vein, and prior radiotherapy involving the liver [224].

NCCN guidelines for mCRC consider RE with Ytrrium-90 as an option of treatment in highly selected patients, with resistant/refractory QT and predominant LMCRC which are not optimally resectable. The ESMO guidelines state that RE with Yttrium-90 can prolong PFS in patients with LMCRC failing the available QT [5]. The Spanish guidelines about LMCRC also propose that RE with Yttrium-90 is a chance of treatment when all available treatments fail. In addition, the Spanish guidelines think that RE with Yttrium-90 may be useful as a neoadjuvant treatment when R0 resection of LMCRC is difficult but the volume, outflow, inflow and biliary drainage of hepatic parenchyma is adequate [12].

### 5.4. Other Hepatic Arterially Directed Therapies

Bland embolization uses inert particles of several sizes and composition to obstruct tumor microvasculature, so it leads to tumor infarction. Bland embolization is effective in the treatment of hepatocellular carcinoma and liver metastases of neuroendocrine tumors, but it is not used for LMCRC [225].

## 6. Emerging Technologies and Future Directions

### 6.1. Emerging Technologies

As we have reviewed previously, an adequate targeting and the tumor and its margins is very important, as it is one of the most important prognostic factors. This targeting is highly dependent on the skill and experience of the operator. Needle navigation systems as well as ablation planning software are limited to viewing these datasets on 2D monitor screens. Emerging technologies in software allow 3D reconstructions that show promising results [226]. Taking a step further is the development and adaptation of virtual reality and augmented reality. This allows to stereoscopically view 3D datasets in actual three-dimensional space, resulting in improved spatial understanding as well as procedure execution and safety, especially for inexperienced operators [227,228].

Although improvements in navigation will allow to precisely target the tumor, many of the ablative techniques discussed below do not allow to monitor changes in the treated tissue, and the evaluation of the ablated zone has to be done post-procedurally. Understanding and predicting the expected ablation zone for a single device is complex and involves interactions among the settings chosen by the operator, tissue density, tissue hydration, and local heat sinks, as discussed earlier [229]. In this regard, ablation planning software is an emerging technology that will help to provide better care to our patients [230].

Electrochemotherapy results from the combination of IRE with concomitant administration of chemotherapeutic drugs. Bleomycin is the agent used in the main trials published with this technique. Encouraging results are found with complete responses ranging within 55–85% with no serious adverse events [231,232,233]. Further studies are needed before applying electrochemotherapy in routine clinical practice.

Y90 radiation segmentectomy for LMCRC could be an option of treatment in selected patients. Padia et al. treated 36 patients who were not candidates for surgical resection or thermal ablation with LM (11 patients had LMCRC) by Y90 radiation segmentectomy; ORR was 92% (28% partial response, 64% stable disease) and adverse events were low, without hepatic-related toxicity [234]. Kurilova et al. treated 10 patients with ≤ 3 LM and limited treatment options. Y90 radiation segmentectomy can provide a 2-year local tumor control rate of 83% [235].

### 6.2. Liver Transplantation in CRC

The International Hepato-Pancreato-Biliary Association commissioned an international multidisciplinary group of experts to develop consensus guidelines, named the Liver Transplantation for Colorectal liver Metastases 2021 (LT-CoMet 21) working group, and this guideline was published recently. This guideline provides initial standardization of terms and proposes a therapeutic algorithm in a very novel oncology field as the transplant oncology. The selection of patients is crucial, as patients candidates for transplant must have non-resectable disease limited to the liver with favorable molecular prognosis markers (patients with V600 BRAF mutant CRC are excluded, due to their poor prognosis, while high microsatellite instability and deficient DNA mismatch repair are also excluded, but due to the notable results of immunotherapy in this population and concerns about the high allograft rejection rate associated with the administration of immunotherapy after solid organ transplantation). Adequate response to systemic therapy must be documented for at least 6 months over an interval of at least 1 year from the diagnosis of non-resectable colorectal liver metastases, ongoing at the time that the transplant is planned. In accordance with the ethical principle of utility, which incorporates beneficence and non-maleficence, this stringent patient selection process aims to identify patients with non-resectable colorectal liver metastases who would derive the most survival benefit from liver transplantation. Nonetheless, the inclusion of patients with malignant indications in transplantation list will cause tension to the principles of utility and justice, which is recognized by theLT-CoMet 21 working group [236].

Previous clinical trials in liver transplantation in LMCRC are the SECA I and SECA II trials, which showed promising results. In SECA I trial, 21 unresectable patients without signs of extrahepatic disease and a minimum of 6 weeks of chemotherapy treatment were included. No adjuvant chemotherapy was administered after the transplantation. The Kaplan–Meier estimate of 5-year OS was 60%. Risk factors for death were carcinoembryonic antigen (CEA) > 80 mg/L, progressive disease on chemotherapy, size of largest lesion > 5.5 cm, and less than 2 years from resection of the primary tumor to transplantation. [237]. In the SECA II trial, with the stricter selection criteria and patients with significantly better prognostic factors than patients in SECA-I, the OS was improved to 83%. Disease-free survival rates at 1, 2, and 3 years were 53%, 44%, and 35%, respectively. Overall survival rates from time of relapse at 1, 2, and 4 years were 100%, 73%, and 73%, respectively. Regarding prognostic factors, the Fong Clinical Risk Score of 1 to 2 at the time of diagnosis resulted in longer disease-free survival than a score of 3 to 4 [238].

## 7. Role of Biomarkers

RAS mutations are probably the most studied genetic alterations influencing prognosis in patients with CRC. Mutations in the RAS family of proto-oncogenes (KRAS, NRAS, HRAS) are present in 30–45% of patients with colorectal cancers. These mutations cause a constitutive activation of the MAPK pathway, leading to resistance to treatment with EGFR antibodies.

RAS mutations affecting prognosis have been better defined in the resectable population, but efforts were made recently to characterize their effect in the ablation setting. As we have discussed earlier, RAS mutations in codons 12, 13 61 and 146 and NRAS mutations in codons 12, 13 and 61 have shown to be a negative prognostic factor of recurrence [56,62,210]. The interaction between RAS mutations and the margin of resection was also studied. Calandri et al. showed that achieving minimal ablation margins > 10 mm in mutant RAS tumors provides similar 3-year LTPFS as achieving ≤ 10 mm ablation margins in wild-type RAS tumors [51]. Shady et al. showed that mutant RAS tumors carry a risk of progression 15.6-fold higher when compared with wild-type RAS tumors ablated with ablation margins of ≥ 6 mm [56]. Although the RAS mutational status is not considered a contraindication to image-guided ablation, some authors recommend that RAS-mutated patients be considered candidates for resection only if adequate margins can be obtained (3D minimal ablation margins of ≥ 5 mm and ≥ 10 mm are desirable) [239].

BRAF, a protein kinase in the mitogen-activated protein kinase (MAPK) signaling pathway, is another important prognostic factor in CRC. BRAF V600E mutation are identified in 5–8% of patients, with especially poor prognosis with a median survival of only 12 months with chemotherapy alone [240]. Patients with CRC with BRAF V600E mutations rarely have metastases limited to the liver, and those who undergo liver resection (1–6.1%) often develop disease recurrence [241]. To our knowledge, no study has addressed the role of BRAF V600E mutations in population treated with local ablation therapies or with liver-directed therapies.

Prognostic relevance of microsatellite instability (MSI) with deficient DNA mis- match repair (dMMR) is well established in early-stage tumors but its role in the metastatic setting is still under debate [242]. The promising results of first-line immunotherapy as shown by the KEYNOTE-177 trial with long-term disease control and response rate (43.8%), higher than the chemotherapy arm, encourage the use of immunotherapy in the neoadjuvant setting [243]. However, published studies have focused on the neoadjuvant setting but in early stages and locally advanced cancers. The NICHE trial showed 100% pathological response in dMMR and 27% in pMMR in early-stage CRC when using neoadjuvant ipilimumab and nivolumab [244]. Toripalimab, an anti PD-1 inhibitor, also showed 88% complete responses in this setting [245]. However, concerns in the use of immunotherapy in the conversion therapy scenario were raised as progressive disease, as the best response, was present in 29.4% of patients in the immunotherapy group in contrast to 12.4% in the chemotherapy arm. Ongoing trials are exploring the combination of the anti-PD1 antibodies nivolumab and pembrolizumab with SIRT and TACE (NCT03380130, NCT03033446, NCT02837029, NCT03099564, and NCT03143270) and with radiotherapy or ablation (NCT02437071).

## 8. Discussion

The treatment of LMCRC is a clear example of advances in oncology that illustrates a change in a classic oncology idea (local therapy being used for local disease and systemic therapy being used in patients with systemic disease). LMCRC represent systemic disease, but the use of local therapies, such as surgical resection or ablation for patients with resectable LMCRC, may be a curative treatment [4]. Systemic therapy is the conventional treatment in patients with unresectable LMCRC. The benefit of non-surgical local treatments in patients with limited disease naturally raises the possibility that liver-directed regional therapies may provide benefit in patients with more extensive LMCRC.

**Figure 1:**
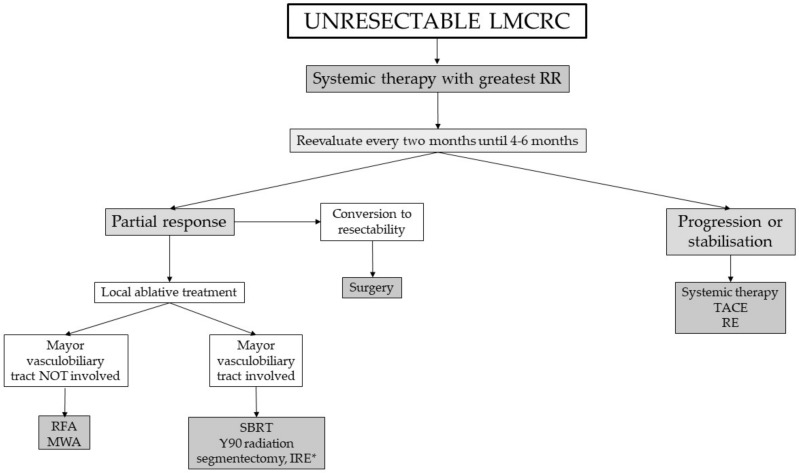
Algorithm treatment of LMCRC. Adapted from [5,19]. Abbreviations: IRE—irreversible electroporation, MWA—microwave ablation, SBRT—stereotactic body radiotherapy, TACE—transarterial chemoembolization, RE—radioembolization, RFA—radiofrequency ablation. * Y90 radiation segmentectomy is indicated for tumors near major bile ducts or vascular structures.

Compared to chemotherapy alone, the addition of a multidisciplinary approach with local and loco-regional treatments improves outcomes in candidates for a more radical therapy. However, we must take into consideration that this subset of patients represents a group with an intrinsically better prognosis than the patients who are not candidates for intensive therapy and are only candidates for chemotherapy. Guidelines from ESMO, for example, consider that one of the first steps in the evaluation of a metastatic patient is to categorize it into a “never resectable” or “potentially resectable” group that will guide the therapies that the patient will receive [5,19]. The Figure 1 shows our suggestion of the algorithm treatment in unresectable LMCRC.

Results reported with diverse LAT are difficult to compare to those reported for surgery, giving the inherent selection bias present in these series. The lack of randomized, prospective data of local ablative therapies is reflected in the consensus guidelines from different societies. All guidelines share the consideration that for a resectable patient, the *gold standard* treatment is surgery, and while LAT is traditionally reserved for patients with medical, technical or personal contraindications to surgery, this notion is being challenged in recent guidelines, as LAT can provide survival and local control rates similar to those of surgery in well-selected patients. No technique is preferred, as there is little evidence comparing different treatments, and the decision for using a certain technique is often related to the expertise of the treating team or operator. Different meta-analyses and systematic reviews have contributed to more robust evidence compared to isolated prospective series, but still the evidence is considered to be weak, and data from randomized, phase III trial are needed. Therefore, the COLLISION trial results are awaited, as they will shed some light on the best treatment for resectable patients.

Regarding loco-regional treatments, the heterogeneity of the studies makes it difficult to interpret and compare the results between them. Many of these studies have a limited number of patients or are conducted at single institutions. Several studies that use one non-surgical treatment present diverse inclusion criteria, use different definitions, apply diverse techniques and local or systemic treatment, or evaluate the response to treatment in a different way. There are treatments that are not used in some places; for example, CI is used in European countries in an exceptional way, but it is frequent in United States. All this generates the impossibility of having robust results, and there are many questions in medical oncology that do not have an answer today, including the following: Which ablative therapy is better? Which embolization treatment is more useful? Is DEBIRI better than RE with Yttrium-90? It is necessary to conduct powerful clinical trials that try to respond to these clinical questions, although this may not be practical, given the complexities in non-surgical clinical treatments. Clinicians should discuss the non-surgical treatment with patients, taking into account patient preferences and the uncertainty of the evidence.

The selection of patients who could benefit from non-surgical local treatments is an interesting challenge in oncology. A small group of patients with LMCRC are candidates for these therapies. The majority of patients with LMCRC have some disease outside the liver, such as of the lung or peritoneum. The pattern of progression in patients with LMCRC is variable and not easy to predict, so clinical studies are necessary to identify patients with good prognosis. Other relevant consideration is the age of the patients. This raises the question of whether elderly patients could benefit from non-surgical treatment of LMCRC. Life expectancy is increasing in Western society, and many patients of LMCRC are diagnosed at an advanced age [246].

The advances in the treatment of LMCRC show the importance of working in a multidisciplinary way. The patients with LMCRC should be evaluated by a team composed of medical oncologists, surgical oncologists, radiation oncologists and interventional radiologists. This requires that all specialists speak in a similar way; in other words, the definition of unresectable, resectable and possible or potential resectable patient with LMCRC have to be similar, and every specialist has to know every non-surgical therapy, their indications and their effectiveness.

## 9. Conclusions

The management of LMCRC is becoming more aggressive and complex. New non-surgical treatment of LMCRC complements systemic therapy by providing an opportunity for the local control of hepatic spread. Actual evidence supports non-surgical treatment in LMCRC, but it can be useful to maximize disease control and survival in selected patients. Although a multidisciplinary approach is recommended, it is necessary to establish standard practices for non-surgical treatment in LMCRC.

## Figures and Tables

**Table 1:** Types of non-surgical local treatments in liver metastases of colorectal cancer.

Local Treatments	
Thermal	Radiofrequency ablation (RFA) Microwave ablation (MWA) Cryoablation Laser interstitial thermal therapy (LITT) * High-intensity focused ultrasound (HIFU) *
Non-thermal	Radiotherapy (SBRT) Irreversible electroporation (IRE) *
**Loco-Regional Treatments**	
Embolization	Bland particulate embolization * Chemoinfusion or hepatic artery infusion Transarterial chemoembolization Radioembolization

* Laser interstitial thermal therapy (LITT), high-intensity focused ultrasound (HIFU) and bland particulate embolization have not been used in large scale for LMCRC.

**Table 2:** Main trials of conversion chemotherapy.

Study	Year	*n*	Study Population	Treatment	RR	Liver Resection Rate
CELIM [25]	2010	106	No molecular selection	FOLFOX6/FOLFIRI + Cet	All patients: 63% KRAS ex2wt: 70%	33%
GONO [26]	2010	30	No molecular selection	FOLFOXIRI + Bev	80%	40%
BOXER [27]	2011	46	No molecular selection	CAPOX + Bev	78%	40%
Ye et al. [23]	2013	138	KRAS exon 2 wild-type	FOLFIRI/FOLFOX ± Bev	57% vs. 29%	26% vs. 7%
OLIVIA [28]	2015	80	No molecular selection	FOLFOXIRI + Bev vs. FOLFOX + Bev	81% vs. 62%	49% vs. 23%
PLANET-TTD [24]	2017	77	KRAS exon 2 wild type	FOLFOX + Pmab vs. FOLFIRI + Pmab	74% vs. 67%	34% vs. 46%
VOLFI [29]	2019	99	KRAS exon 2 wild type. Other RAS mutations included but excluded from the analysis	FOLFOXIRI + Pmab vs. FOLFOXIRI	87.3% vs. 60.6%	Global population: 33.3% vs. 12.1% Convertible population: 75% vs. 36.4%.

Abbreviations: Bev—bevacizumab, CAPOX—capecitabine + oxaliplatin, Cet—cetuximab, FOLFIRI—5-FU + irinotecan, FOLFOX—5-FU + oxaliplatin, FOLFOXIRI—5-FU + oxaliplatin + irinotecan, *n*—number of patients, Pmab—panitumumab, RR—resection rate.

**Table 3:** Representative retrospective studies of RFA and MWA.

Study	Year	*n*	Treatment	Indication of Treatments	OS (Months)	PFS/RFS (Months)	Local Control
Abdalla et al. [88]	2004	468	Group 1: Intraoperative RFA, surgery ± RFA Group 2: CT	RFA reserved for patients not candidates for surgery. CT: Not candidates for RFA or surgery	S: 3-year 73% *p* < 0.05 S + RFA: 3-year 43% RFA: 3-year 37%	S: Better than RFA and RFA + surgery *p* < 0.05	Liver recurrence S: 11% *p* < 0.05 RFA + surgery: 28% *p* < 0.05 RFA: 84% *p* < 0.05
Kim et al. [89]	2011	505	Surgery, surgery + RFA, RFA only (open or percutaneous)	RFA reserved for patients not candidates for surgery.	S: 5-year: 34.6% S + RFA: 5-year: 22.9% RFA: 5-year: 14.3%	S: 5-year DFS: 16.2% S + RFA: 5-year DFS: 18.4% RFA: 5-year DFS 7% *p* < 0.05	NR
Schiffman et al. [90]	2010	140	Surgery, RFA only (open)	RFA reserved for patients not candidates for surgery. Solitary metastases.	S: 112.7 m RFA: 50.2 m *p* < 0.05	S: 52 m RFA: 42m	Local recurrence S: 12.6% RFA: 35.6% *p* < 0.05
Tinguely et al. [91]	2020	727	Surgery, MWA only (open, laparoscopic and percutaneous)	MWA reserved for patients not candidates for surgery with lesions < 3 cm	WTPS S: 54.5 m WTPS RFA: 43.4 m *p* < 0.05 WPS S: 54.7 m WPS MWA: 48 m	NR	NR
Shady et al. [36]	2016	165	RFA only (percutaneous)	RFA reserved for patients not candidates for surgery or recurrence of previous surgery	mOS; 36 m Patients without LP: 65 m Patients locally retreated after LP: 51 months Patients not treated after LP: 22 m *p* < 0.05	NR	mLTPFS: 26 m. Poor prognosis: tumors > 3 cm, ablation margin <5 mm *p* < 0.05
Shady et al. [55]	2018	154	RFA only, MWA only,	RFA reserved for patients not candidates for surgery	NR	NR	24-m LTPFS: RFA: 66% 24-m LTPFS MWA: 60% LTP < 5 mm AM: 71% *p* < 0.05 LTP 5–10 mm AM: 14.8% LTP 10 mm AM: 0%
Dijkstra et al. [75]	2021	136	RFA only, MWA only, surgery	Recurrent CLM. RFA and MWA use at discretion of a MDT	S: 49.4 m Ablative: 54.4 m	Distant 3-year PFS S: 26.6% Distant 3-year PFS ablative: 24%	1-year LTPFS S: 96.1% 1-year LTPFS ablative: 91.6%

Abbreviations: AM—ablation margin, CLM—colorectal liver metastases, DFS—disease-free survival, LP—local progression, M—months, MDT—multidisciplinary team, mLTPFS—median local tumor progression-free survival, mOS—median overall survival, *n*—number of patients, OS—overall survival, PFS—progression-free survival, S—surgery, WPS—with propensity score matching, WTPS—without propensity score matching. Significant differences between groups are represented by significative *p* values. *p* values of not significant differences between arms are not shown.

**Table 4:** Main clinical trials using RFA, MWA or cryoablation as experimental arm.

Study	Type	Year	*n*	Treatment	Complications	OS (Months)	PFS (Months)	Local Control
CLOCC [67]	RCT	2012	119	Local + systemic Systemic only	Total percentage of patients not reported.	8 years: 35.9% 8 years: 8.9%	16.8 9.9	Hepatic progression: 46.7% Hepatic progression: 78%
ARF2003 [68]	Phase II	2012	52	RFA ± surgery	RFA: 42.3% RFA + surgery: 40.4%	5 years: 43.9%	1 year: 27%	1 y LPFS: 46%
Shibata et al. [80]	RCT	2000	30	MWA Surgery	14.28% 12.5%	27 m 25 m	11.3 (DFS) 13.3 (DFS)	Not reported
Korpan et al. [98]	RCT	1997	123	Cryosurgery (including cryoablation) Conventional surgery	10% 20%	10 years: 81% 10 years: 92%	NR	10 years: 14% 10 years: 5%

Abbreviations: DFS—disease-free survival, LPFS—local progression-free survival, MWA—microwave ablation, *n*—number of patients, NR—not reported, OS—overall survival, RCT—randomized controlled trial, RFA—radiofrequency ablation, Y—year. No significant differences between groups are noted; therefore, *p* value is not shown.

**Table 5:** Main trials using CI in unresectable LMCRC.

Comparing CI to Systemic QT							
Study	Type	Year	*n*	Treatment	ORR (%)	mOS (Months)	
MSKCC [154]	RCT	1987	48 51	CI FUDR IV FUDR	53 21 (*p* < 0.05)	17 12	
Martin [155]	RCT	1990	61 76	HAI FUDR IV 5-FU	48 21 (*p* < 0.05)	12.6 10.5	
Kerr [156]	RCT	2003	145 145	HAI 5-FU+LV IV 5-FU+LV	22 19	14.7 14.8	
CALGB [159]	RCT	2006	68 67	HAI FUDR+LV IV 5-FU	47 24 (*p* < 0.05)	24.4 20 (*p* < 0.05)	
**Comparing CI with Systemic QT**							
**Study**	**Type**	**Year**	***n***	**CI**	**IV QT**	**ORR (%)**	**CTR (%)**
D’Angelica [164]	Phase II	2015	49	FUDR	Oxaliplatin/irinotecan/bevacizumab or FOLFIRI/bevacizumab	76	47
Levi [165]	Phase II	2016	64	Irinotecan/oxaliplatin/5-FU	Cetuximab	40.6	29.7
Lim [166]	Multicenter retrospective	2017	61	Oxaliplatin	5-FU/LV or 5-FU/Bev or 5-FU/anti-EGFR	21.3	16.4
Pak [167]	Phase II	2018	64	FUDR	Oxaliplatin/irinotecan or FOLFIRI/bevacizumab	73	52

Abbreviations: 5-FU—fluorouracil, Bev—bevacizumab, CI—chemoinfusion, CTR—conversion to resection, FUDR—floxuridine, IV QT—intravenous chemotherapy (systemic therapy), LV—leucovorin, mOS—median of overall survival, *n*—number of patients, NR—not reported, ORR—objective responses rates, RCT—randomized controlled trial.

**Table 6:** Results of studies evaluating benefits of addition of DEBIRI to systemic therapy in first-line treatment of LMCRC.

Conventional TACE [192]						
Study Arm		mOS (Months)		5-Year PFS (%)	Conversion to Resection (%)	ORR (%)
QT		17.5		2.5	7.0	11.6
QT + TACE		28.4		22.3	30.8	46.2
QT + cetuximab		18.9		7.6	10.5	34.2
QT + cetuximab + TACE		30.3		20.3	32.4	44.1
**DEBIRI [193]**						
**Study Arm**	***n***	**mOS (Months)**	**mPFS (Months)**	**RECIST** **Response Rate (%)**	**Choi Response Rate (%)**	**Toxicity** **(% Grade 3, 4)**
mFOLFOX + Bev	30	NR	15	2 m: 89% 4 m: 95% 6 m: 89%	82	46
mFOLFOX + Bev + DEBIRI	40	NR	12 (*p* = 0.18)	2 m: 88% 4 m: 97% 6 m: 92% (All NS)	98 (*p* = 0.01)	54

Abbreviations: Bev—bevacizumab, DEBIRI—drug eluting beads loaded with irinotecan, FOLFOX—5-fluoruracil, leucovorin, oxaliplatin, mFOLFOX—modified FOLFOX, mPFS—median of progression-free survival, mOS—median of overall survival, QT—chemotherapy, RECIST—response evaluation criteria in solid tumor, TACE—transarterial chemoembolization.

**Table 7:** Results of studies evaluating benefits of addition of DEBIRI to systemic therapy in second-line or later treatment of LMCRC [193].

Study	Study Type	Study Arm or Arms	*n*	mOS (Months)	mPFS (Months)	ORR (%)	Toxicity (% Grade 3, 4)
Aliberti [194]	SA	DEBIRI	82	25	8	NR	25
Di Noia [195]	SA	DEBIRI + capecitabine	40	8	4	17.5	15
Fiorentini [196]	RCT	FOLFIRI	38		4	20	Neutropenia: 44 vs. 4 Mucositis: 20 vs. 1
		DEBIRI	36	Longer in DEBIRI arm (*p* = 0.031)	7 (*p* = 0.006)	68.6 (*p* = NR)	

Abbreviations: DEBIRI—drug eluting beads loaded with irinotecan, FOLFOX—5-fluoruracil, leucovorin, oxaliplatin, FOLFIRI—5-fluoruracil, leucovorin, irinotecan, mPFS—median of progression-free survival, mOS—median of overall survival.

**Table 8:** Results of study evaluating benefits of addition of RE with Yttrium-90 to systemic therapy in first-line treatment of LMCRC [208].

Study Arm	*n*	mOS (Months)	mPFS (Months)	ORR (%)	Toxicity (% Grade 3, 4, 5)
mFOLFOX	549	23.3	10.3	63	OR 1.42, 95% CI: 1.09 to 1.85, *p* = 0.089
FOLFOX + RE	554	22.6 (*p* = 0.061)	11.3 (*p* = NS)	72 (*p* = 0.0012)	

Abbreviations: CI—confidence interval, FOLFOX—5-fluoruracil, leucovorin, oxaliplatin, mFOLFOX—modified FOLFOX, mPFS—median of progression-free survival, mOS—median of overall survival, OR—odds ratio, ORR—overall response rate, RE—radioembolization with Yttrium-90.

## Abbreviations

5-FU	5-fluorouracil
AE	Adverse events
Bev	Bevacizumab
CI	Confidence interval
CI	Chemoinfusion
CRC	Colorectal cancer
CR	Complete response
CRS	Clinical risk score
CT	Computer tomography
CTR	Conversion to resection
D	Dimension
DEBIRI	Drug eluting beads loaded with Irinotecan
DFS	Disease-free survival
dMMR	Mismatch-repair deficient
ECG	Electrocardiogram
EGFR	Epidermal growth factor receptor
ESMO	European Society of Medical Oncology
FFLP	Freedom from local progression
FOLFIRI	5-fluorouracil, leucovorin, irinotecan
FOLFOX	5-fluorouracil, leucovorin, oxaliplatin
FOLFOXIRI	5-fluouracil, leucovorin, oxaliplatin, irinotecan
FUDR	Floxuridine
HCC	Hepatocellular carcinoma
HR	Hazard ratio
IL-2	Interleukin 2
IRE	Irreversible electroporation
HIFU	High-intensity focused ultrasound
LAT	Local ablative therapies
LMCRC	Liver metastases of colorectal cancer
LPFS	Local progression-free survival
LT-CoMet 21	Liver Transplantation for Colorectal liver Metastases 2021
LTP	Local tumor progression
LV	Leucovorin
mCRC	Metastatic colorectal cancer
MDT	Multidisciplinary team
mOS	Median overall survival
mPFS	Median progression-free survival
MR	Magnetic resonance
MSI	Microsatellite instability
MWA	Microwave ablation
NCCN	National Comprehensive Cancer Network
NR	Not reported
OR	Odds ratio
ORR	Overall response rate
OS	Overall survival
PFS	Progression-free survival
PVA	Polyvinyl alcohol
QT	Chemotherapy
RBOC	Radioembolization Brachytherapy Oncology Consortium
RCT	Randomized controlled trial
RE	Radioembolization
RECIST	Response evaluation criteria in solid tumors
RFA	Radiofrequency ablation
RFS	Recurrence-free survival
RILD	Radiation-induced liver disease
SBRT	Stereotactic body radiotherapy
SPRECT	Single photon emission computed tomography
TAVE	Transarterial chemoembolization
US	Ultrasound
VEGF	Vascular endothelial growth factor
